# Estimating the cost of HIV services for key populations provided by the LINKAGES program in Kenya and Malawi

**DOI:** 10.1186/s12913-023-09279-w

**Published:** 2023-04-04

**Authors:** Marjorie Opuni, Jorge Eduardo Sanchez-Morales, Jose Luis Figueroa, Andrea Salas-Ortiz, Louis Masankha Banda, Alice Olawo, Spy Munthali, Julius Korir, Meghan DiCarlo, Sergio Bautista-Arredondo

**Affiliations:** 1 Independent Researcher, Geneva, Switzerland; 2 Division of Health Economics and Health Systems Innovations, National Institute of Public Health (INSP), Cuernavaca, Mexico; 3 FHI 360, Lilongwe, Malawi; 4 FHI 360, Nairobi, Kenya; 5 University of Malawi, Zomba, Malawi; 6 Kenyatta University, Nairobi, Kenya; 7 FHI 360, Durham, NC USA

**Keywords:** Costs, Health economics, HIV, Kenya, Malawi, Key populations

## Abstract

**Background:**

Data remain scarce on the costs of HIV services for key populations (KPs). The objective of this study was to bridge this gap in the literature by estimating the unit costs of HIV services delivered to KPs in the LINKAGES program in Kenya and Malawi. We estimated the mean total unit costs of seven clinical services: post-exposure prophylaxis (PEP), pre-exposure prophylaxis (PrEP), HIV testing services (HTS), antiretroviral therapy (ART), sexually transmitted infection (STI) services, sexual and reproductive health (SRH) services, and management of sexual violence (MSV). These costs take into account the costs of non-clinical services delivered alongside clinical services and the pre-service and above-service program management integral to the LINKAGES program.

**Methods:**

Data were collected at all implementation levels of the LINKAGES program including 30 drop-in-centers (DICs) in Kenya and 15 in Malawi. This study was conducted from the provider’s perspective. We estimated economic costs for FY 2019 and cost estimates include start-up costs. Start-up and capital costs were annualized using a discount rate of 3%. We used a combination of top-down and bottom-up costing approaches. Top-down methods were used to estimate the costs of headquarters, country offices, and implementing partners. Bottom-up micro-costing methods were used to measure the quantities and prices of inputs used to produce services in DICs. Volume-weighted mean unit costs were calculated for each clinical service. Costs are presented in 2019 United States dollars (US$).

**Results:**

The mean total unit costs per service ranged from US$18 (95% CI: 16, 21) for STI services to US$635 (95% CI: 484, 785) for PrEP in Kenya and from US$41 (95% CI: 37, 44) for STI services to US$1,240 (95% CI 1156, 1324) for MSV in Malawi. Clinical costs accounted for between 21 and 59% of total mean unit costs in Kenya, and between 25 and 38% in Malawi. Indirect costs—including start-up activities, the costs of KP interventions implemented alongside clinical services, and program management and data monitoring—made up the remaining costs incurred.

**Conclusions:**

A better understanding of the cost of HIV services is highly relevant for budgeting and planning purposes and for optimizing HIV services. When considering all service delivery costs of a comprehensive HIV service package for KPs, costs of services can be significantly higher than when considering direct clinical service costs alone. These estimates can inform investment cases, strategic plans and other budgeting exercises.

**Supplementary Information:**

The online version contains supplementary material available at 10.1186/s12913-023-09279-w.

## Background

Together with their partners, key populations (KPs) at higher risk of HIV infection—including men who have sex with men (MSM), sex workers (SW), people who inject drugs (PWID), and transgender women (TGW)—are estimated to comprise 65% of new HIV infections globally [1]. In sub-Saharan Africa, new infections among KPs and their partners make up 72% of new HIV infections in West and Central Africa and 32% in Southern and Eastern Africa and there is growing concern that as HIV epidemics in the general population are better controlled, the relative importance of KPs will increase [1–3]. Forty years since the first cases of AIDS were reported, KPs continue to be marginalized and criminalized in many countries, HIV prevention services for KPs continue to be inaccessible to many, and providing HIV testing and treatment services to KPs living with HIV remains a challenge even in countries with high performing HIV programs [1]. To achieve HIV epidemic control, countries around the world, including in sub-Saharan Africa, will need to significantly scale up effective HIV services for KPs [2–4].

Global guidance on HIV services for KPs describes a package of clinical services—many of which are the same services as for the general population [5]. However, effective HIV services for KPs differ in important ways from HIV services for general populations. To address the challenges and barriers often faced by KPs, clinical services for KPs need to be implemented interdependently with structural interventions that address stigma, discrimination, and violence [6–8]. In addition, besides service-level activities, effective KP HIV services also comprise important efforts below- and above-service level [9–11]. KPs often require substantial outreach conducted by peers in the community to facilitate service utilization and retention in care [12–14]. Effective KP programs include differentiated service delivery models and community-based and community-led approaches to service delivery [5, 15]. Likewise, community organization staff and volunteers often need substantial support, including program management support, technical assistance, training, and oversight. Moreover, pre-service delivery activities, including population mapping and size estimation, are integral to the effective delivery of KP HIV services [16–18]. Critically, these non-clinical features are viewed as core elements of comprehensive HIV services for KPs and not as optional extras [4].

To scale up HIV services for KPs, additional resources will need to be allocated to these services. To date, KP HIV services globally have been significantly under-funded with expenditures on KP HIV services much lower than would be expected given the burden of HIV among KPs [19, 20] and representing only a fraction of estimated resource needs [21]. Developing investment cases for additional resources and allocating available resources efficiently across HIV services requires accurate and timely information on the costs of HIV services. Though there is a growing literature on the costs of HIV services for general populations in low- and middle-income countries, data remain scarce on the costs of HIV services for KPs and these mostly focus on the delivery of clinical services alone [22]. We are aware of only one costing study of a KP HIV program that considered the costs of clinical and non-clinical service elements, including interventions required to reach and engage KPs and project management [10].

The objective of this study was to bridge this gap in the literature by estimating the unit costs of the HIV services delivered to KPs in the Linkages Across the Continuum of HIV Services for Key Populations Affected by HIV (LINKAGES) program in Kenya and Malawi. LINKAGES was led by FHI 360 and funded by the United States President’s Emergency Plan for AIDS Relief (PEPFAR) from 2014 to 2021. Implemented in over 30 countries in Africa, Asia, and the Caribbean, the program provided a comprehensive package of services to KPs with the goal of reducing HIV transmission among KPs and their sexual partners and improving KP enrollment and retention in HIV treatment services. We estimated the unit costs of the following clinical HIV services delivered to KPs as part of LINKAGES in Kenya and Malawi: post-exposure prophylaxis (PEP), pre-exposure prophylaxis (PrEP), HIV testing services (HTS), antiretroviral therapy (ART), sexually transmitted infection (STI) services, sexual and reproductive health (SRH) services, and management of sexual violence (MSV). Our unit cost estimates also reflect the cost of non-clinical services delivered alongside these clinical services (peer outreach, structural interventions that address stigma, discrimination, and violence, KP empowerment) and the pre-service (KP mapping and size estimation) and above-service program management and data monitoring that was an integral part of the LINKAGES program.

## Methods

### Study setting

Kenya and Malawi have generalized HIV epidemics with concentrated sub-epidemics among KPs [23]. In 2021, HIV prevalence in 15–49-year-old adults was estimated to be 4% in Kenya [24] and 7.7% in Malawi [25]. Recent HIV prevalence data for FSW and MSM in Kenya are not available [24]. In Malawi, the latest HIV prevalence for FSW and MSM was estimated to be 49.9% and 12.9% [25]. KPs face important structural barriers in both countries that impact their vulnerability to HIV infection and impede their access to health services [26]. A recent cross-country analysis of law, criminalization, and HIV describes sex work as criminalized in both countries and same-sex sexual acts as criminalized in Kenya and partially criminalized in Malawi [26].

### Program description

In each country, operationalization of LINKAGES involved an initial start-up phase with pre-service delivery activities. Subsequently, a comprehensive package of KP HIV services was scaled up with activities executed at multiple implementation levels (Figure S1). LINKAGES program headquarters provided overall program guidance and technical assistance. LINKAGES country offices provided on-the-ground program management and technical support. Local community-based organizations, including KP-led organizations, referred to as implementing partners (IPs) delivered HIV services to KPs. Services were delivered in communities through outreach activities and at drop-in centers (DICs)—sites where KPs received HIV services, met with peers, and conducted social and community mobilization activities.

The LINKAGES program included the following core program areas based on global guidance [5, 27–30]: 1) engage KPs in population size estimation, mapping, and program planning; 2) KP empowerment and engagement; 3) structural interventions; 4) peer outreach; 5) clinical services including PEP, PrEP, HTS, ART, STI services, SRH services, MSV, and condom and lubricant promotion and distribution; 6) program management; and 7) monitoring and data use. These core program areas were subdivided into program elements, spelling out the interventions carried out (Figure S2). All programmatic work and technical assistance were organized along these program areas and elements.

### Study sample

Our study sample corresponded to the multi-level LINKAGES program implementation structure and included 30 DICs in Kenya and 15 DICs in Malawi; 18 IPs in Kenya and two IPs in Malawi; two LINKAGES country offices (one per country); and LINKAGES program headquarters. Of the 30 DICs in Kenya, 11 DICs provided services to FSW only, three to MSW only, two to MSM only, and 14 DICs provided services to FSW, MSW, and MSM. Of the 15 DICs in Malawi, 11 DICs provided services to FSW and four to MSM and TGW. In Kenya, we included all IPs and DICs that were part of the LINKAGES program in FY 2019. In Malawi, one IP was excluded (along with its four DICs).

### Data collection

This costing study was implemented following the Global Health Cost Consortium guidelines [31]. We developed costing frameworks for each country to capture the LINKAGES program activities at all implementation levels. Our frameworks mapped activities and associated inputs and outputs to the seven program areas mentioned above. Costing frameworks were developed for the start-up years (FY 2015 and FY 2016 for Malawi and FY 2016 for Kenya) and for FY 2019. Based on these costing frameworks, we developed standardized Excel-based tools to collect information comparable across implementation levels in the two countries. Data were collected retrospectively for the start-up years and prospectively for FY 2019. Cost data were obtained from financial reports, payroll records, program manager reports, facility consumption data reports, program expense files, and asset registers. Additional data from headquarters and country offices were extracted from expenditure records provided by LINKAGES program headquarters. We collected monthly data on quantities and prices for seven input categories: staff, clinical supplies, utilities and operations, transportation, equipment, training, and external services (Table S1). Corresponding output data were obtained from databases in IPs and DICs including number of people treated with PEP, number of people on PrEP, number of people tested for HIV, number of people on ART, number of people screened for STIs, number of people provided a contraceptive method, and number of people provided post-gender-based violence care. For each staff member in country offices, IPs, and DICs, we collected estimates of the proportion of time spent on each of the seven program areas and on each of the clinical services through questionnaires.

### Costing approach

This costing study was conducted from the provider’s perspective (i.e., the LINKAGES program). We estimated economic costs, which include the value of all resources used in the program, including those for which there was no financial transaction, such as donated goods. Annual costs were estimated with service delivery costs calculated for U.S. Government (USG) fiscal year (FY) 2019 (October 1, 2018 to September 30, 2019). Our cost estimates also include start-up costs incurred prior to service delivery. Start-up and capital costs were annualized using a discount rate of 3% and capital goods were assumed to have a useful life of 10 years [31]. We used a combination of top-down and bottom-up costing approaches to estimate unit costs of clinical HIV services delivered to KPs [10, 32]. Top-down methods were used to estimate headquarters, country office, and IP costs and allocate them to DICs. Bottom-up methods were used to measure the quantities and prices of all inputs used to produce services in DICs. We allocated all DIC-level costs to estimate site-level costs of clinical services (PEP, PrEP, HTS, ART, STI services, SRH services, MSV, and condom and lubricant promotion and distribution). All costs are presented in 2019 US dollars (US$). Country costs for FY 2019 were converted from local currencies to US$ using mid-year exchange rates for 2019 (Kenya: 102.01 Kenyan shillings and Malawi: 739.46 Malawian kwacha).

Further details on the methods and allocation weights used to distribute above-service level (headquarters, country office, IP) and start-up costs to DICs can be found in Table S2. In summary, headquarters and country office costs for FY2019 were distributed equally across DICs. Start-up costs were annualized over ten years using a 3% discount rate and the FY 2019 allocation of start-up costs was distributed equally across DICs. For the 13 IPs with only one DIC, IP costs were allocated to the corresponding DIC. For the seven IPs with multiple DICs, the allocation approach used was a function of the input: IP costs of staff, recurrent inputs, and equipment were allocated proportionally across DICs; transportation and training costs, which were available only at the IP level, were distributed across DICs based on DIC staff time weights. We used bottom-up methods to estimate the DIC costs of clinical supplies, staff, peer workers, other recurrent inputs, and equipment, multiplying the number of inputs used with input prices. Total LINKAGES program costs per DIC were obtained by aggregating above-service level allocations to DICs with DIC-level costs.

We used the following approach to estimate the unit cost of PEP, PrEP, HTS, ART, STI, SRH, and MSV services from these DIC-specific total LINKAGES program costs (Table S3). We separated costs into direct and indirect input costs. Direct input costs included the costs of clinical staff and peer workers and the costs of clinical supplies. Costs of clinical staff and peer workers were allocated to each clinical service based on the time allocation they reported. Similarly, clinical supplies were allocated to each clinical service based on resource use. For all indirect inputs shared across services (non-clinical staff, utilities, external services, equipment, training, transportation, headquarters, country office, and start-up), we weighted input costs using allocation weights derived by combining the annual number of clients per service over the total annual number of clients in the DIC and the proportion of staff and peer worker time dedicated to each service (Table S3). The proportion of clinical services provided in each DIC was used to distribute the costs of condom and lubricant promotion and distribution to the other clinical services provided, as no output data were available for this clinical service. We present volume-weighted mean unit costs for each service, which were calculated as the sum of total service costs across all DICs in a country divided by the sum of service outputs across all DICs in the country [33].

Given the centrality of the program areas to the LINKAGES program, we also assessed the breakdown of the overall unit costs of PEP, PrEP, HTS, ART, STI, SRH, and MSV services across the LINKAGES program areas: 1) engage KPs in population size estimation, mapping, and program planning; 2) KP empowerment and engagement; 3) structural interventions; 4) peer outreach; 5) clinical services; 6) program management; and 7) monitoring and data use. As described in more detail in Table S4, we separated inputs into two categories: those for which the program area was identified in the data collection tool (clinical and non-clinical staff, peer workers, clinical supplies, transportation, and training) and those for which the program area was unspecified (utilities, external services, equipment, headquarters, country office, and start-up). Unspecified inputs were allocated according to program area staff time weights (Table S4). Average country-level program area staff time weights were then used to distribute the unit costs per service across LINKAGES program areas.

We bootstrapped all unit costs reported 100 times to estimate standard errors and calculate 95% confidence intervals.

## Results

Table 1 shows the number of clinical services provided in DICs in Kenya and Malawi during FY 2019. All clinical services were delivered in both countries except for PEP, which was only provided in Kenya. Though most DICs in Kenya provided PrEP, only three DICs in Malawi did so. In both countries, only DICs serving FSW delivered SRH services. Almost all DICs in both countries provided HTS, ART, STI, and MSV services. The vast majority of services delivered in both countries were STI services and HTS. In Kenya, STI screenings and HIV tests each comprised 46% of all services DICs delivered by DICs. In Malawi, STI screenings and HIV tests made up 73% and 17% of all services provided. The number of services and visits for each clinical service varied substantially by DIC in both countries. Facilities in Kenya tended to have significantly more clients than those in Malawi for all services except SRH services.

**Table 1:** Clinical services provided by LINKAGES program drop-in centers in Kenya and Malawi, FY 2019

**Country**	**DIC**	**KP served**	**PEP**	**PREP**	**HTS**	**ART**	**STI**	**SRH**	**MSV**
			**Treated**	**On PREP**	**Tested**	**On ART**	**Screened**	**Provided CM**	**Provided post-GBV care**
Kenya	A01	FSW	114	38	5,577	338	4,212	577	752
	D02	FSW	11	59	1,169	33	1,335	21	17
	D04	FSW	56	30	2,618	195	2,712	42	47
	E01	FSW	26	4	3,123	161	3,155	127	33
	E02	FSW	2	0	2,087	168	1,956	61	0
	F01	FSW	66	2	2,789	132	3,134	232	13
	L01	FSW	9	16	2,186	79	2,216	0	92
	O01	FSW	0	3	750	78	768	203	7
	O02	FSW	0	1	514	56	525	40	5
	P01	FSW	7	5	3,945	127	4,030	312	263
	R06	FSW	9	63	1,194	13	1,670	245	14
	D01	FSW_MSW_MSM	0	41	3,234	70	3,509	57	218
	D03	FSW_MSW_MSM	15	73	1,805	66	2,034	28	84
	E03	FSW_MSW_MSM	24	3	1,590	81	1,485	15	8
	H01	FSW_MSW_MSM	25	166	6,606	315	6,880	103	44
	H02	FSW_MSW_MSM	0	18	1,628	36	1,547	49	17
	J01	FSW_MSW_MSM	11	16	2,708	143	2,196	526	70
	K01	FSW_MSW_MSM	33	16	3,830	89	2,664	106	386
	M01	FSW_MSW_MSM	10	5	2,528	198	2,565	0	212
	N01	FSW_MSW_MSM	2	1	1,582	70	1,542	13	284
	R01	FSW_MSW_MSM	3	30	2,430	124	2,768	121	55
	R02	FSW_MSW_MSM	4	66	3,557	152	3,834	134	95
	R03	FSW_MSW_MSM	8	66	2,420	75	2,694	89	95
	R04	FSW_MSW_MSM	4	54	2,629	79	2,721	196	12
	R05	FSW_MSW_MSM	28	38	2,033	102	2,126	174	42
	C01	MSM	34	27	1,029	198	1,192	0	10
	G	MSM	6	0	1,422	79	1,597	0	17
	B01	MSW	45	185	3,705	329	4,236	0	332
	I01	MSW	2	45	1,839	158	2,191	0	43
	Q01	MSW	0	16	914	13	794	0	28
		TOTAL	554	1,087	73,441	3,757	74,288	3,471	3,295
Malawi	B01	FSW	0	9	480	82	3,379	280	18
	B02	FSW	0	6	437	80	4,071	343	16
	B03	FSW	0	7	831	124	3,295	978	18
	B04	FSW	0	0	1,045	172	2,339	161	37
	B05	FSW	0	0	552	86	912	75	23
	B06	FSW	0	0	378	97	2,438	183	24
	B07	FSW	0	0	453	106	1,951	248	21
	B08	FSW	0	0	492	81	1,298	54	20
	B09	FSW	0	0	348	100	1,508	536	22
	B10	FSW	0	0	521	43	1,681	12	22
	B11	FSW	0	0	724	105	4,325	322	27
	A01	MSM_TG	0	0	793	6	3,054	0	1
	A02	MSM_TG	0	0	473	12	653	0	7
	A03	MSM_TG	0	0	328	8	1,471	0	4
	A04	MSM_TG	0	0	508	5	3,284	0	3
		TOTAL	0	22	8,363	1,107	35,659	3,192	263

FY 2019 refers to U.S. Government fiscal year from October 1, 2018 to September 30, 2019, *FSW* female sex workers, *MSW* male sex workers, *MSM* men who have sex with men, *TGW* transgender women, *DIC* drop-in center, *PEP* post-exposure prophylaxis, *PrEP* pre-exposure prophylaxis, *HTS* HIV testing services, *ART* antiretroviral therapy, *STI* sexually transmitted infections, *SRH* sexual and reproductive health, *MSV* management of sexual violence, *CM* contraceptive method, *GBV* gender-based violence. The number of services provided during the year is displayed for PEP, HTS, STI services, SRH services, and MSV. For PrEP and ART, annual visits are shown, regardless of the number of services an individual received during the year

The volume-weighted mean unit costs for the clinical services implemented in Kenya and Malawi are shown in Table 2. For each service, Table 2 also shows the breakdown of the mean total unit cost into the following components: direct clinical service costs, condom and lubricant promotion and distribution costs, and indirect costs. Given the small number of outputs, we did not calculate the unit cost for PrEP in Malawi. When looking at the overall unit cost, the mean unit cost per clinical service ranged from US$18 (95% CI: 16, 21) for STI services to US$635 (95% CI: 484, 785) for PrEP in Kenya and from US$41 (95% CI: 37, 44) for STI services to US$1,240 (95% CI 1156, 1324) for MSV in Malawi. Considering the direct clinical service unit cost, the mean unit cost per clinical service ranged from US$2 (95% CI: 2, 3) for STI to US$162 (95% CI: 125, 198) for ART in Kenya and from US$9 (95% CI: 7, 10) for STI services to US$362 (95% CI: 242, 367) for MSV in Malawi. Clinical costs, including both direct clinical service costs and the costs of condom and lubricant promotion and distribution, accounted for between 21% (MSV) and 59% (ART) of unit costs in Kenya, and between 25% (MSV) and 38% (ART) in Malawi. Total unit costs were lower in Kenya than they were in Malawi and the difference was especially large for MSV services.

**Table 2:** Volume-weighted mean unit costs per clinical service in Kenya and Malawi, LINKAGES program, FY 2019 in US$ 2019

**Country**	**Clinical service**	**Indicator**	**N**	**Direct clinical service**		**Condoms and lubricants**		**Indirect**		**Total unit cost**	
				**UC**	**95% CI**	**UC**	**95% CI**	**UC**	**95% CI**	**UC**	**95% CI**
**Kenya**	**PEP**	**Treated**	25	95	76–114	5	1–9	356	295–416	456	350–562
	**PREP**	**On PrEP**^a^	30	127	90–165	51	27–74	456	350–562	635	484–785
	**HTS**	**Tested**	30	5	4–6	5	3–7	14	12–16	24	21–27
	**ART**	**On ART**^a^	30	162	125–198	54	29–79	152	132–172	368	298–438
	**STI**	**Screened**	30	2	2–3	5	3–6	11	10–12	18	16–21
	**SRH**	**Provided CM**	23	15	13–18	7	2–12	73	66–80	95	72–117
	**MSV**	**Provided post-GBV care**	29	13	10–17	6	1–11	71	56–86	90	69–110
**Malawi**	**HTS**	**Tested**	15	25	19–30	3	1–4	57	46–67	84	73–94
	**ART**	**On ART**^a^	15	260	177–344	26	14–38	471	407–535	757	608–907
	**STI**	**Screened**	15	9	7–10	2	1–2	30	27–34	41	37–44
	**SRH**	**Provided CM**	11	31	27–35	1	1–2	75	66–85	108	70–145
	**MSV**	**Provided post-GBV care**	15	305	242–367	2	1–3	933	897–969	1240	1156–1324

FY 2019 refers to U.S. Government fiscal year from October 1, 2018 to September 30, 2019, *UC* unit cost, *95% CI* 95% confidence interval, *N* number of drop-in centers, *PEP* post-exposure prophylaxis, *PrEP* pre-exposure prophylaxis, *HTS* HIV testing services, *ART* antiretroviral therapy, *STI* sexually transmitted infections, *SRH* sexual and reproductive health, *MSV* management of sexual violence, *CM* contraceptive method, *GBV* gender-based violence. Given the small number of outputs, the unit cost was not calculated for PrEP in Malawi.^a^For PrEP and ART, unit costs represent the annual cost per person on PrEP and ART

Table 3 displays the breakdown of the total volume-weighted mean unit costs shown in Table 2 by LINKAGES program area. These program areas do not line up perfectly with the unit cost components shown in Table 2. However, they provide additional insight into the relative importance of start-up costs, other KP interventions implemented with clinical services, and above-service level support. The clinical service program area accounted for the largest proportion of total mean unit cost for most services. However, this represented between 20% (MSV) and 54% (ART) of total mean unit costs in Kenya and between 24% (MSV) and 34% (ART) of the costs in Malawi. Program management and monitoring and data use accounted for between 27% (ART) and 42% (SRH and MSV services) of mean unit costs in Kenya and between 31% (PrEP) and 40% (STI services) in Malawi. The KP interventions implemented alongside clinical services (peer outreach, structural interventions, and KP empowerment) accounted for between 15% (ART) and 34% (PEP) of total mean unit costs in Kenya and between 25% (ART) and 31% (MSV) in Malawi. KP mapping and size estimation accounted for between 3% (ART) and 5% (SRH and MSV services) of costs in Kenya and between 6% (PrEP, HTS, ART) and 7% (STI, SRH, and MSV services) in Malawi.

**Table 3:** Distribution of volume-weighted mean unit costs per clinical service by LINKAGES program area, by country, FY 2019 in US$ 2019

**Country**	**Clinical service**	**Indicator**	**N**	**KP mapping & size estimation**		**KP empowerment**		**Structural interventions**		**Peer outreach**		**Clinical services**		**Program management**		**Monitoring & data use**	
				**UC**	**95% CI**	**UC**	**95% CI**	**UC**	**95% CI**	**UC**	**95% CI**	**UC**	**95% CI**	**UC**	**95% CI**	**UC**	**95% CI**
**Kenya**	**PEP**	**Treated**	25	19	16–23	25	21–30	22	18–25	107	89–125	102	85–119	118	98–138	62	52–73
	**PREP**	**On PrEP**^a^	30	28	22–35	36	27–45	31	23–38	127	97–157	161	122–199	167	127–206	85	65–105
	**HTS**	**Tested**	30	1	1–1	1	1–1	1	1–1	3	3–3	9	8–11	6	5–6	3	2–3
	**ART**	**On ART**^a^	30	11	9–13	14	11–17	12	10–14	31	25–37	201	163–239	66	53–78	34	27–40
	**STI**	**Screened**	30	1	1–1	1	1–1	1	1–1	2	2–2	6	6–7	5	4–5	2	2–3
	**SRH**	**Provided CM**	23	4	4–5	5	5–6	5	4–5	20	17–22	21	18–23	26	23–29	14	12–16
	**MSV**	**Provided post-GBV care**	29	4	3–5	5	4–7	5	4–6	20	15–25	18	14–22	25	19–31	13	10–16
**Malawi**	**HTS**	**Tested**	15	5	5–6	5	4–5	3	3–4	13	12–15	27	24–31	19	16–21	11	10–13
	**ART**	**On ART**^a^	15	48	39–58	42	34–51	28	22–33	118	95–142	258	207–309	164	132–197	99	80–119
	**STI**	**Screened**	15	3	3–3	3	2–3	2	2–2	6	6–7	11	10–12	10	9–11	6	6–7
	**SRH**	**Provided CM**	11	7	7–8	6	5–7	4	3–4	20	17–22	32	28–36	22	19–24	17	14–19
	**MSV**	**Provided post-GBV care**	15	86	81–92	75	70–81	49	46–52	261	243–279	298	278–318	291	271–310	179	167–192

FY 2019 refers to U.S. Government fiscal year from October 1, 2018 to September 30, 2019, *UC* unit cost, *95% CI* 95% confidence interval, *N* number of drop-in centers, *PEP* post-exposure prophylaxis, *PrEP* pre-exposure prophylaxis, *HTS* HIV testing services, *ART* antiretroviral therapy, *STI* sexually transmitted infections, *SRH* sexual and reproductive health, *MSV* management of sexual violence, *CM* contraceptive method, *GBV* gender-based violence. Given the small number of outputs, the unit cost was not calculated for PrEP in Malawi. ^a^For PrEP and ART, unit costs represent the annual cost per person on PrEP and ART

**Fig. 1:**
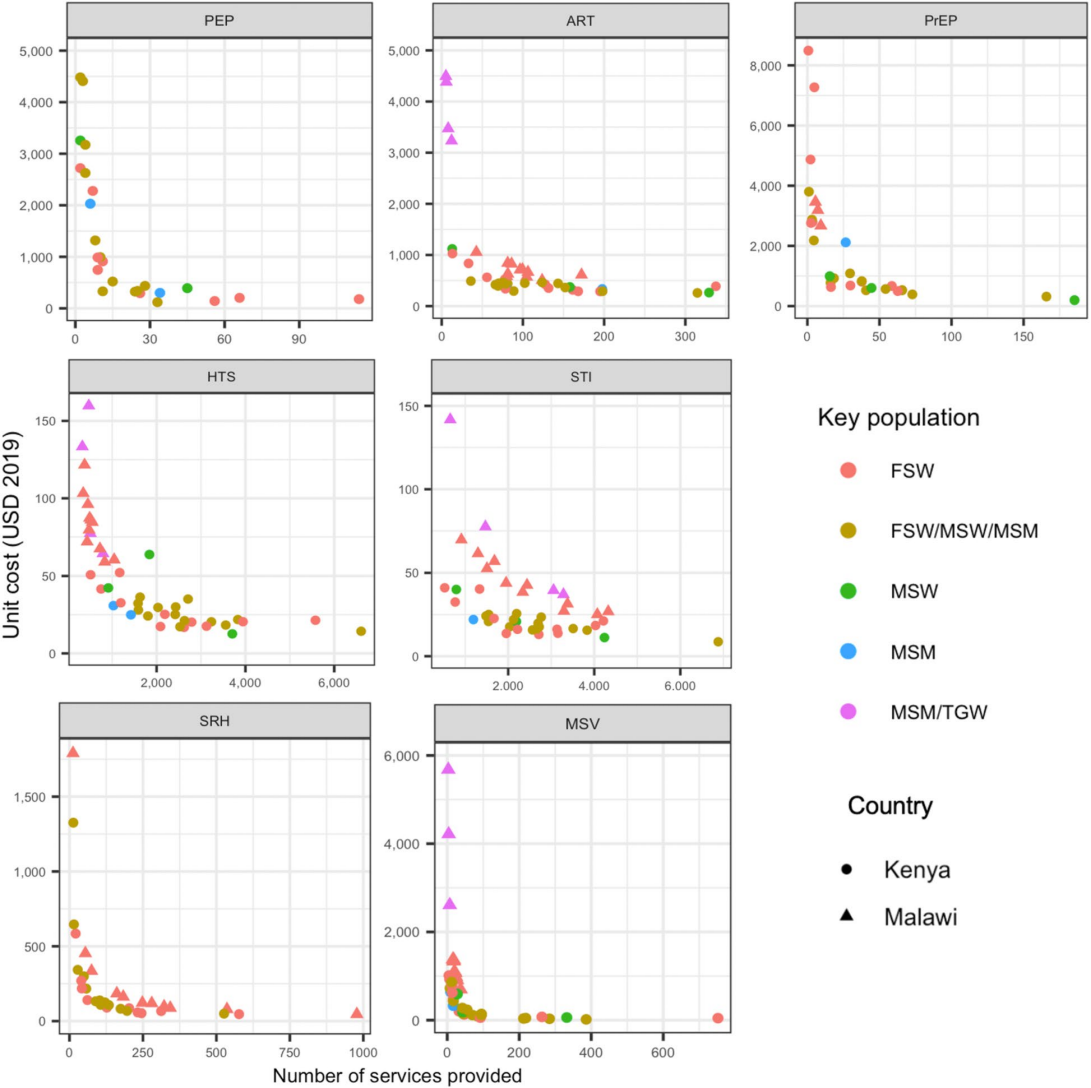
Unit costs per clinical service and numbers of services provided by LINKAGES program drop-in centers in Kenya and Malawi, FY 2019, US$ 2019. Notes: FY 2019 refers to U.S. Government fiscal year from October 1, 2018 to September 30, 2019; *FSW*  female sex workers, *MSW*  male sex workers, *MSM*  men who have sex with men, *TGW* transgender women, *PEP* post-exposure prophylaxis, *PrEP* pre-exposure prophylaxis, *HTS* HIV testing services, *ART* antiretroviral therapy, *STI* sexually transmitted infections, *SRH* sexual and reproductive health, *MSV* management of sexual violence. For PrEP and ART, unit costs represent the annual cost per person on PrEP and ART

Figure 1 shows scatterplots of the relationship between total unit cost and number of services provided for each clinical intervention across DICs (see data in Table S5). The figure shows that for all services, there was a great deal of variation in unit costs in DICs within and between countries. Overall, unit costs tended to be lower at sites providing larger numbers of services consistent with the existence of economies of scale, though the association between unit cost and number of services provided varied across services and was weaker for STI services. DICs in Malawi tended to have fewer clients than those in Kenya for most services and the highest unit costs for ART, HTS, and MSV services were in the DICs in Malawi that provided the fewest services––mostly DICs serving MSM/TGW. Even at comparable volumes of services delivered, unit costs tended to be higher in DICs in Malawi, especially for ART, STI, and SRH services.

## Discussion

This study describes the costs of PEP, PrEP, HTS, ART, STI, SRH, and MSV services delivered to KPs as part of the LINKAGES program in Kenya and Malawi. We found that mean total unit costs for PEP and PrEP were US$356 and US$456 in Kenya. Across the two countries, total unit costs for HTS, ART, STI, SRH, and MSV services ranged between US$24-US$84, US$368-US$757, US$18-US$41, US$95-US$108, and US$90-US$1240 respectively. Direct clinical service costs accounted for between 21 and 59% of total mean unit costs in Kenya, and between 25 and 38% of unit costs in Malawi. Our results show that in comprehensive HIV programs for KPs, considering direct clinical service costs alone grossly underestimates the cost of service delivery to KPs.

Our assessment of clinical service unit costs by LINKAGES program area shows that above-service program management and data monitoring and non-clinical services delivered to KPs alongside clinical services––deemed to be essential by experts––made up large components of service costs [4]. Program management and data monitoring accounted for between 27–42% (Kenya) and 31–32% (Malawi) of mean unit costs for the seven clinical services. KP interventions implemented alongside clinical services (peer outreach, structural interventions that address stigma, discrimination, and violence, and KP empowerment) accounted for between 18–34% (Kenya) and 25–31% (Malawi) of total service unit costs.

We found differences in mean unit costs between the two countries with the mean unit cost for everything except condoms lower in Kenya than in Malawi. Comparing the two countries is difficult, even though the costing methods applied in both countries were the same. We know that in both Kenya and Malawi, KPs face important structural barriers that impact their vulnerability to HIV infection and impede their access to health services [26]. However, from the information available, it is difficult to ascertain the heterogeneity in the levels of stigma, discrimination, and violence against KPs between the two countries. In general, organizations in Kenya delivered significantly more services than those in Malawi. This was true for almost all service categories, and it was especially true for MSV which had the largest difference in unit cost. The differences in service volumes across DICs likely plays an important role in cost differences in the two countries and these could be due to overall differences in site maturity [34]. Another factor contributing to the differences in cost between the two countries is that headquarter and country office costs were proportionally higher in Malawi. Summaries of LINKAGES program achievements in Kenya and Malawi suggest that the program in Malawi may have had a larger portfolio of above-service level activities playing key roles in national HIV policy and guideline development [35, 36].

We also found important differences in unit costs across facilities within countries. This variation suggests that efficiency of service provision might be improved in certain DICs. Our mapping of DIC service unit costs against numbers of services provided suggests the potential for economies of scale as DICs expand the volume of services delivered, although we recognize that there may be a tension between large volume increases and the provision of services to marginalized and hard to reach populations. It is also possible that there are economies of scope and that DICs that provide more categories of services are more efficient than DICs providing fewer categories of services [37, 38]. Finally, there could be differences in the management of DICs that account for some of the variation in unit costs [39]. Though these issues are beyond the scope of this paper, we will explore the relationships between unit cost and service scale, scope, and facility management in future analyses.

We are not aware of previous studies that have captured the unit costs of a combination of clinical services delivered to KPs in Kenya or Malawi. However, we can compare the mean clinical-service unit costs of HTS, ART, and PrEP to those of previous studies conducted in Kenya and Malawi. Our direct clinical service unit costs of US$5 for HTS in Kenya is similar to previous estimates [40, 41] though our unit costs of US$162 and US$127 for ART and PrEP are slightly lower than earlier studies [42–47]. Our direct clinical service unit costs of US$25 for HTS and US$260 for ART in Malawi are somewhat higher than those of previous studies [48–53]. Of course, comparisons across cost studies should be done with caution as input utilization and costs vary over time and methods differ across studies.

The only other costing study of a comprehensive HIV program for KPs that we are aware of is the costing study of the Avahan program in India [4, 10]. Cost analysis of the Avahan program also considered non-clinical service elements including interventions to reach and engage FSW, MSM, and TGW. Like our study, the Avahan costing study also captured the full costs of program implementation including service-level and above-service level costs. The Avahan program components differ and are difficult to compare with those of the LINKAGES program and the scope of the clinical services provided in the Avahan program was more limited focusing mostly on the provision of STI services. It is however possible to compare the proportion of program management and monitoring across the two studies. The proportion of program management and monitoring we found in this study was consistent with the 37% program management component in the costing study of the Avahan program [10].

Given the decline in HIV resources in recent years, there is a growing urgency to understand the cost of HIV services to make sure that HIV resources can be allocated effectively and efficiently [21, 54]. This study provides detailed information on the costs of seven HIV services provided to KPs in Kenya and Malawi. These data together with information on the costs of other HIV services including those for the general population, data on the effectiveness of interventions, as well as epidemiological and demographic data, can be used to inform investment cases, strategic plans, and other budgeting exercises. One of the strengths of this study is that the data analyzed are the result of a unique collaboration with the LINKAGES program in which all levels of the LINKAGES program made data available. The study also benefited from the underlying costing frameworks we developed for each country which mapped out activities and associated inputs and outputs and captured all the activities undertaken at all levels of program implementation.

The following limitations should be kept in mind when considering our findings. We used allocation weights based on staff time allocation to allocate above-service level costs to DICs, to allocate indirect input costs to clinical services, and to allocate unspecified costs to LINKAGES program areas. Staff time allocation in our study was based on self-reported time which can be less reliable when staff activities are irregular [31]. We were unable to use time motion direct observation which is generally viewed as the gold standard though this method is not without limitations (i.e., bias from the observer effect) [31, 55]. Confidentiality concerns in settings with significant stigma, discrimination, and violence against KPs as well as resource constraints made it impossible for us to directly observe staff providing services to KPs. One possibility for future studies on the cost of KP services might be to use text messaging to collect information on current activity for a representative sample of providers, days, and hours. Another limitation was that no information was available on the outputs for peer outreach, structural interventions, KP empowerment, for example, and the overlap between these outputs and the outputs of each of the clinical services. An additional challenge was that we used routine monitoring data to capture information on outputs. Though the LINKAGES program had a reasonably robust system for data reporting and verification, the level of detail, quality, and completeness of routine data varied across IPs and DICs. The documentation of donated goods also varied across IPs and DICs and some misreporting of in-kind contributions is possible. Finally, we acknowledge that this study focuses on the costs of services provided to KPs as part of the LINKAGES program in Kenya and Malawi and we do not capture the costs of any referrals from DICs to public health facilities. Similarly, we do not capture information on the costs of HIV services provided to KPs who access services directly from public health facilities.

## Conclusions

A better understanding of the cost of HIV services is highly relevant for budgeting and planning purposes and for optimizing HIV services. This descriptive analysis of the costs of HIV services for KPs in Kenya and Malawi shows that when considering all service delivery costs of a comprehensive HIV service package for KPs, costs of services can be significantly higher than when considering direct clinical service costs alone. These estimates can be used to inform investment cases, strategic plans and other budgeting exercises in Kenya and Malawi. They can also be used to pressure test the cost of KP services used in planning and budgeting exercises in other countries where no similar data are available. Additional studies on the cost of comprehensive KP services are urgently needed.

## Supplementary Information

**Supplementary information 1: Figure S1.** LINKAGES program implementation levels and sample size by level. **Figure S2.** LINKAGES program areas and elements. **Table S1.** Cost categories and sub-categories included in the LINKAGES costing study. **Table S2.** Methods used to estimate total LINKAGES program cost per DIC. **Table S3.** Approach used to estimate costs per clinical service (PEP, PREP, HTS, ART, STI, SRH, MSV). **Table S4.** Approach used to estimate costs per LINKAGES program area (KP mapping & size estimation, KP empowerment, structural interventions, peer outreach, clinical services, management, monitoring & data use). **Table S5.** Unit costs per clinical service and numbers of services provided by LINKAGES program drop-in centers in Kenya and Malawi, FY 2019, US$ 2019.

## Data Availability

The data generated and/or analyzed during this study are available upon reasonable request.

## Abbreviations

ART: Antiretroviral therapy
DIC: Drop-in-center
FSW: Female sex workers
FY: Fiscal year
HTS: HIV testing services
IP: Implementing partners
KP: Key population
LINKAGES: Linkages Across the Continuum of HIV Services for Key Populations Affected by HIV
MSM: Men who have sex with men
MSV: Management of sexual violence
MSW: Male sex workers
PEP: Post-exposure prophylaxis
PEPFAR: United States President’s Emergency Plan for AIDS Relief
PREP: Pre-exposure prophylaxis
PWID: People who inject drugs
SDG: Sustainable Development Goal
SRH: Sexual and reproductive health
STI: Sexually transmitted infection
SW: Sex workers
TGW: Transgender women
US$: United States dollars
USG: United States Government
