# Supplementary material for: Estimating the cost of HIV services for key populations provided by the LINKAGES program in Kenya and Malawi
Source: BMC Health Serv Res. 2023 Apr 4;23:337. doi: 10.1186/s12913-023-09279-w (PMC10071702; doi:10.1186/s12913-023-09279-w)
Supplement: Supplementary file 1 — Supplementary information 1: Figure S1. LINKAGES program implementation levels and sample size by level. Figure S2. LINKAGES program areas and elements. Table S1. Cost categories and sub-categories included in the LINKAGES costing study. Table S2. Methods used to estimate total LINKAGES program cost per DIC. Table S3. Approach used to estimate costs per clinical service (PEP, PREP, HTS, ART, STI, SRH, MSV). Table S4. Approach used to estimate costs per LINKAGES program area (KP mapping & size estimation, KP empowerment, structural interventions, peer outreach, clinical services, management, monitoring & data use). Table S5. Unit costs per clinical service and numbers of services provided by LINKAGES program drop-in centers in Kenya and Malawi, FY 2019, US$ 2019. [file 12913_2023_9279_MOESM1_ESM.docx]

**Estimating the cost of HIV services for key populations provided by the LINKAGES program in Kenya and Malawi**

**Supplementary information 1**

**Figure S1. LINKAGES program implementation levels and sample size by level**


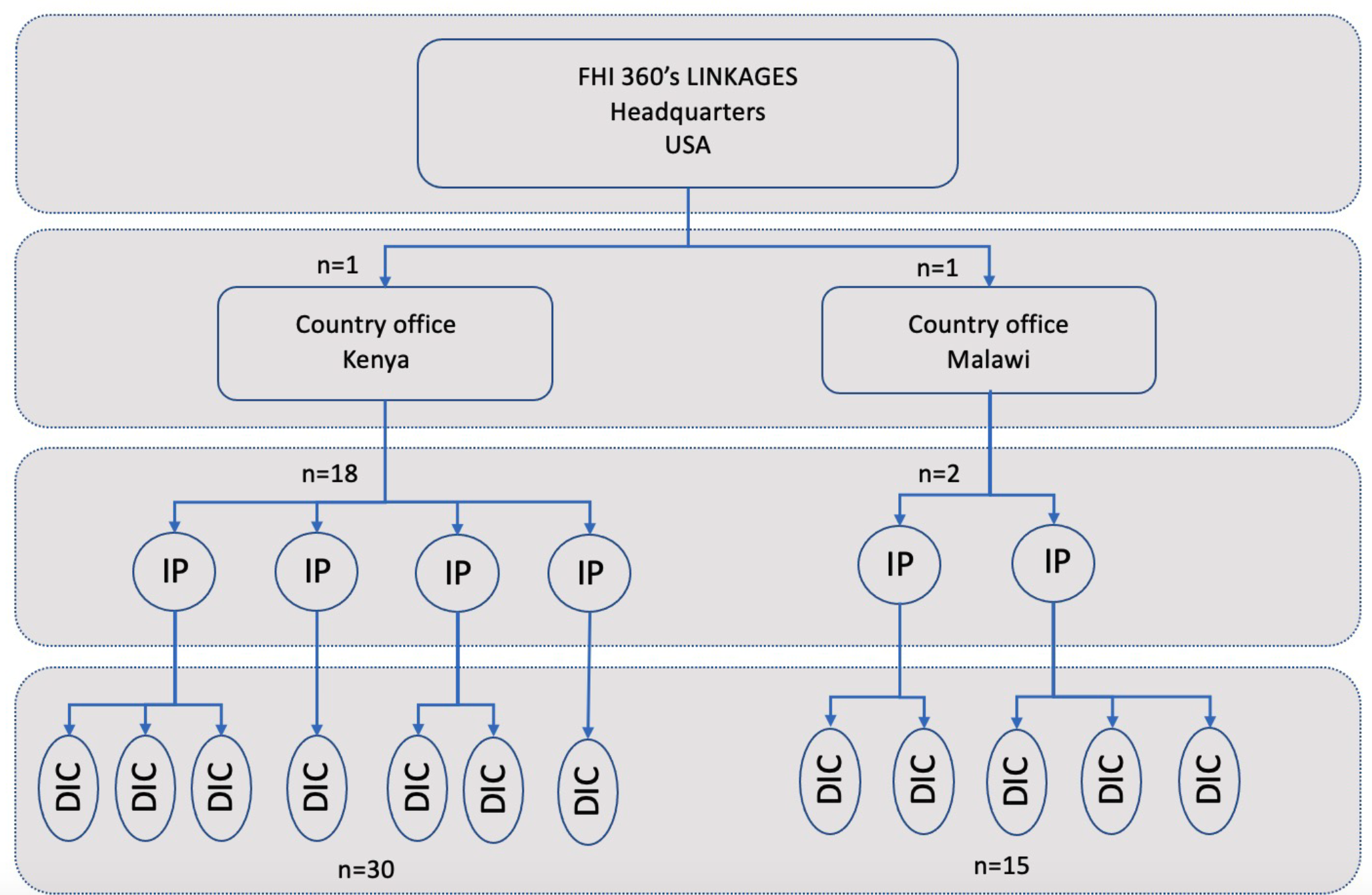


Notes: *LINKAGES* Linkages Across the Continuum of HIV Services for Key Populations Affected by HIV, *IP* implementing partner, *DIC* drop-in center, *n* number of offices/organizations/facilities

**
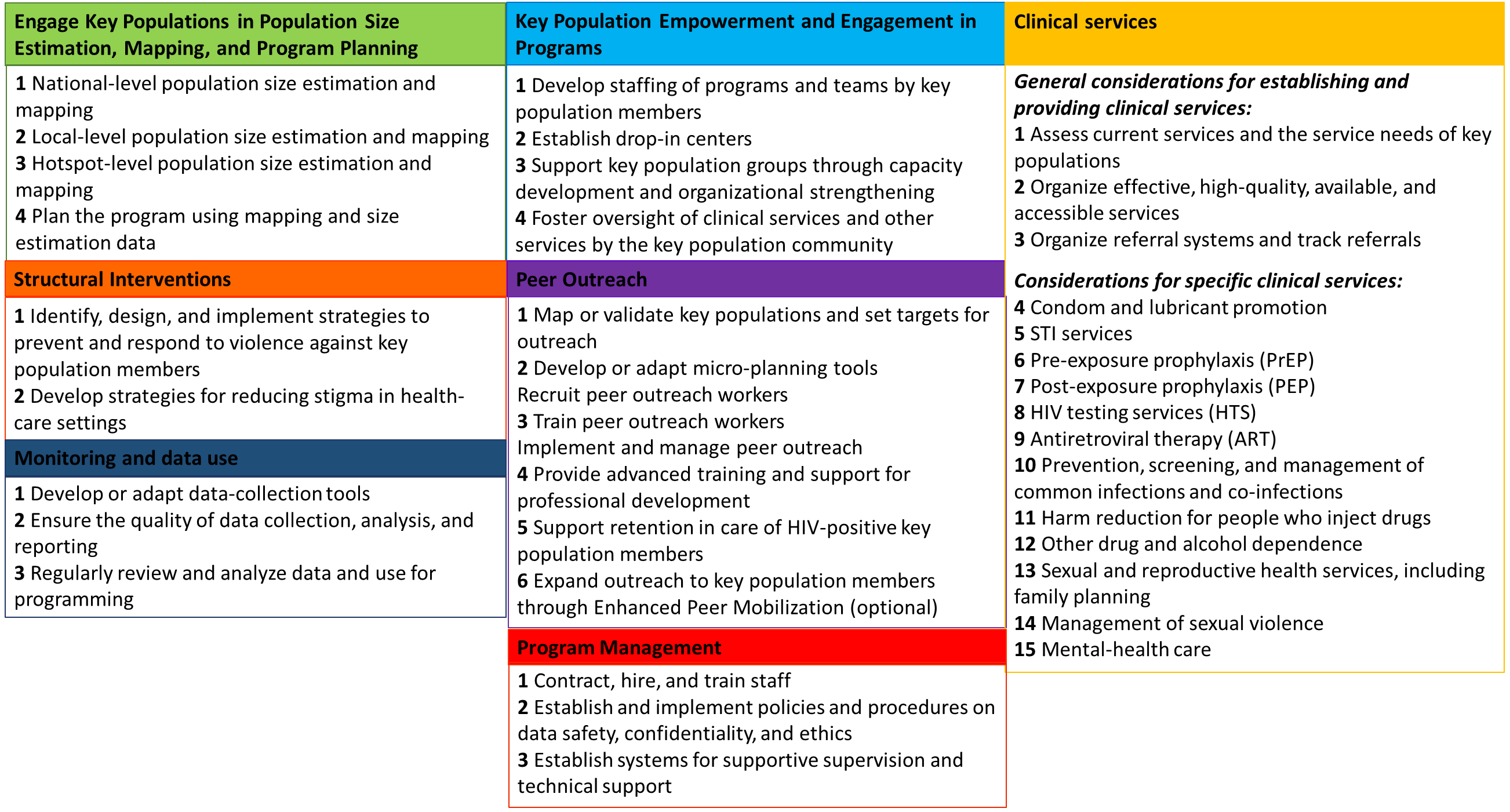
Figure S2. LINKAGES program areas and elements**

Notes: Each program area is contained in a colored rectangle. Associated program elements are listed below each program area. (Source: FHI 360. LINKAGES Kenya FY18 Implementation Plan. 2017)

**Table S1. Cost categories and sub-categories included in the LINKAGES costing study**

| **Cost category** | **Sub-categories** |
| --- | --- |
| **Clinical supplies** | Male condoms, female condoms, lubricants, STI treatment drugs, PEP drugs, PrEP drugs, ART drugs, family planning commodities, HIV and STI test |
| **Staff** | Monthly salary, total benefits package, medical insurance and worker compensation, life insurance, social security, pension fund, overtime pay, severance, paid leave, allowances, accommodation, relocation, benefits, etc. |
| **Peer workers** | Monthly payment (peer educators, peer navigators, outreach workers) |
| **Transportation** | Staff per diem, public transportation, rent of vehicles, fuel, vehicle maintenance, vehicle insurance, flights, general transportation, outreach transportation, supervision transportation |
| **Other recurrent**  **(Utilities, operations, and external services)** | Electricity consumption, water consumption, diesel for generator, oil/paraffin/kerosene, internet connection, telephone service, rent, building maintenance, building insurance, general office supplies, security services |
| **Equipment** | Type of item bought (computing equipment, cabinets, chairs, desks, etc.) |
| **Training** | HIV care & treatment training, outreach workers training, peer educator training, annual review meetings, community advisory meetings, staff training, etc. |
|  | |

Notes: *STI* sexually transmitted infections, *PEP* post-exposure prophylaxis, *PrEP* pre-exposure prophylaxis, *ART* antiretroviral therapy

**Table S2. Methods used to estimate total LINKAGES program cost per DIC**

- Above facility costs (headquarters, country office, implementing partners, and start up) were distributed to individual DICs (top-down approach).
- Bottom-up micro costing methods were used to measure the quantities and prices of inputs used to produce services in DICs.

|  | **Allocation factors** | |  |
| --- | --- | --- | --- |
| **Input description** | **DICs overseen by IP managing only one DIC** | **DICs overseen by IP managing more than one DIC** |  |
| **Startup** |  |  | Top-down approach |
| Startup (expenditures at headquarters and country levels and subawards with no breakdown by input type) | Total cost was equally distributed across DICs | Total cost was equally distributed across DICs |  |
|  |  |  |  |
| **Implementation** |  |  |  |
| *Headquarters* |  |  |  |
| Program expenditures at HQ level (staff and indirect) | Total cost was equally distributed across DICs | Total cost was equally distributed across DICs |  |
| *Country office* |  |  |  |
| Program expenditures at CO level (staff, recurrent, external services, travel, tax, training, capital) | Total cost was equally distributed across DICs | Total cost was equally distributed across DICs |  |
| *Implementing partner (main office)* |  |  |  |
| Staff, utilities, external services, equipment | All costs allocated to corresponding DIC | Allocated proportionally to DICs overseen by IP |  |
| Transportation and training | All costs allocated to corresponding DIC | Allocated based on DIC staff time weights^#^ |  |
| *DIC level costs* |  |  |  |
| Staff, peer workers, clinical supplies, utilities, external services, equipment, transportation and training | Price was multiplied by quantity of each reported input and added to obtain the totals | | Bottom-up approach |

Notes: *CO* country office, *DIC* drop-in center, *HQ* headquarters, *IP* implementing partner

^#^${SLW}_{k}=\frac{\sum_{k=1}^{n} \sum_{i=1}^{n} {TD}_{I,k,IP}}{\sum_{IP=1}^{n} \sum_{k=1}^{n} \sum_{i=1}^{n} {TD}_{i,k,IP}}$

where SLW =service level staff time weight, k=drop-in center, i=individual, IP=implementing partner, TD=time allocation

**Table S3. Approach used to estimate costs per clinical service (PEP, PREP, HTS, ART, STI, SRH, MSV)**

- Total costs per DIC were separated into direct input costs (costs of inputs directly linked to the production of a specific service) from indirect input costs (overhead costs)
- Overhead costs were distributed among the services using weights that combine the allocation of staff time and the number of outputs
- Cost of condoms and lubricants was treated separately. We assumed that they were provided proportionally to the number of services provided

| **Type of cost** | **Input description** | **Allocation factors** |
| --- | --- | --- |
| **Direct costs** | Clinical staff  Peer workers | Cost was distributed among services according to time allocation reported |
|  | Clinical supplies | Cost of each supply used in each service was added |
| **Condoms and lubricant** | Condoms and lubricants | Output-based weights^##^ |
| **Indirect costs** | Non-clinical staff  Utilities and external services  Equipment  Training  Transportation  Startup costs  Headquarters  Country office | Weights based on a combination of outputs and staff time allocation^###^ |

Notes: *DIC* drop-in center, *PEP* post-exposure prophylaxis, *PrEP* pre-exposure prophylaxis, *HTS* HIV testing services, *ART* antiretroviral therapy, *STI* sexually transmitted infections, *SRH* sexual and reproductive health, *MSV* management of sexual violence

^#^${SW}_{nm}=\frac{{FTEs}_{nm}}{\sum{FTEs}_{n}}\times100$

^##^${QW}_{nm}=\frac{Q_{nm}}{\sum Q_{n}}\times100$

^###^${CombW}_{nm}=\frac{{SW}_{nm}+{QW}_{nm}}{2}$

where SW = staff time weight, FTE = full time equivalent, QW = output based weight, Q = output, CombW= combined output and staff time weight n = service provided (PEP, PREP, HTS, ART, STI, SRH, MSV), m=drop-in center

**Table S4. Approach used to estimate costs per LINKAGES program area (KP size estimation, KP empowerment, structural interventions, peer outreach, clinical services, management, monitoring & data use)**

| **Input description** | **Allocation factors** |
| --- | --- |
| *Known program area* |  |
| Clinical and non-clinical staff  Peer workers  Clinical supplies  Transportation  Training | None. In the data collection tool, the allocation by program area was directly identified. |
| *Unspecified program area* |  |
| Utilities and external services  Equipment  Country office  Headquarters  Startup | Weights based on staff time allocation among the seven program area^#^ were used to allocate these costs among the 7 program areas |
| **Costs per clinical service** | **Allocation factors** |
| PEP, PREP, HTS, ART, STI, SRH, MSV | Average program area staff time weights at the country level were used to allocate unit costs per service across program areas |

Notes: LINKAGES program areas: 1) engage KPs in population size estimation, mapping, and program planning; 2) KP empowerment and engagement; 3) structural interventions; 4) peer outreach; 5) clinical services; 6) program management; and 7) monitoring and data use

^#^${PAW}_{PA}=\frac{\sum_{i=1}^{n} {TD}_{i,k,PA}}{\sum_{PA=1}^{7} \sum_{i=1}^{n} {TD}_{i,k,PA}}$

where PAW =program area staff time weight, k=drop-in center, i=individual, PA=program area, TD=time allocation

**Table S5. Unit costs per clinical service and numbers of services provided by LINKAGES program drop-in centers in Kenya and Malawi, FY 2019, US$ 2019**

| **Country** | **DIC** | **KP served** | **PEP** |  | **PrEP** |  | **HTS** |  | **ART** |  | **STI** |  | **SRH** |  | **MSV** |  |
| --- | --- | --- | --- | --- | --- | --- | --- | --- | --- | --- | --- | --- | --- | --- | --- | --- |
|  |  |  | **UC** | **Q** | **UC** | **Q** | **UC** | **Q** | **UC** | **Q** | **UC** | **Q** | **UC** | **Q** | **UC** | **Q** |
| Kenya | A01 | FSW | 178 | 114 | 811 | 37.7 | 21 | 5,577 | 388 | 337.6 | 21 | 4,212 | 46 | 577 | 46 | 752 |
| Kenya | D02 | FSW | 911 | 11 | 668 | 58.6 | 52 | 1,169 | 836 | 33.1 | 40 | 1,335 | 585 | 21 | 478 | 17 |
| Kenya | D04 | FSW | 142 | 56 | 683 | 29.8 | 17 | 2,618 | 288 | 195.0 | 13 | 2,712 | 218 | 42 | 129 | 47 |
| Kenya | E01 | FSW | 293 | 26 | 2,796 | 3.6 | 18 | 3,123 | 318 | 161.3 | 14 | 3,155 | 90 | 127 | 200 | 33 |
| Kenya | E02 | FSW | 2,720 | 2 | 23,025 | 0.3 | 17 | 2,087 | 290 | 168.1 | 14 | 1,956 | 141 | 61 |  |  |
| Kenya | F01 | FSW | 203 | 66 | 4,871 | 2.2 | 20 | 2,789 | 353 | 131.6 | 16 | 3,134 | 58 | 232 | 796 | 13 |
| Kenya | L01 | FSW | 746 | 9 | 633 | 16.3 | 25 | 2,186 | 340 | 78.5 | 16 | 2,216 |  |  | 58 | 92 |
| Kenya | O01 | FSW |  |  | 2,763 | 2.5 | 42 | 750 | 525 | 77.8 | 33 | 768 | 86 | 203 | 927 | 7 |
| Kenya | O02 | FSW |  |  | 8,491 | 0.7 | 51 | 514 | 563 | 55.8 | 41 | 525 | 270 | 40 | 1,012 | 5 |
| Kenya | P01 | FSW | 2,278 | 7 | 7,271 | 4.8 | 20 | 3,945 | 427 | 127.4 | 18 | 4,030 | 67 | 312 | 74 | 263 |
| Kenya | R06 | FSW | 987 | 9 | 499 | 62.7 | 33 | 1,194 | 1,029 | 13.3 | 23 | 1,670 | 53 | 245 | 622 | 14 |
| Kenya | D01 | FSW/MSW/MSM |  |  | 525 | 40.7 | 20 | 3,234 | 448 | 70.3 | 17 | 3,509 | 217 | 57 | 48 | 218 |
| Kenya | D03 | FSW/MSW/MSM | 520 | 15 | 389 | 72.8 | 24 | 1,805 | 419 | 66.2 | 18 | 2,034 | 343 | 28 | 90 | 84 |
| Kenya | E03 | FSW/MSW/MSM | 324 | 24 | 2,874 | 3.2 | 28 | 1,590 | 438 | 81.3 | 24 | 1,485 | 646 | 15 | 725 | 8 |
| Kenya | H01 | FSW/MSW/MSM | 339 | 25 | 316 | 165.8 | 14 | 6,606 | 259 | 314.8 | 9 | 6,880 | 138 | 103 | 148 | 44 |
| Kenya | H02 | FSW/MSW/MSM |  |  | 926 | 18.4 | 36 | 1,628 | 490 | 35.9 | 25 | 1,547 | 299 | 49 | 432 | 17 |
| Kenya | J01 | FSW/MSW/MSM | 331 | 11 | 913 | 16.3 | 35 | 2,708 | 444 | 143.3 | 26 | 2,196 | 50 | 526 | 115 | 70 |
| Kenya | K01 | FSW/MSW/MSM | 119 | 33 | 774 | 16.1 | 22 | 3,830 | 295 | 88.7 | 16 | 2,664 | 109 | 106 | 17 | 386 |
| Kenya | M01 | FSW/MSW/MSM | 994 | 10 | 2,181 | 4.5 | 17 | 2,528 | 291 | 198.1 | 16 | 2,565 |  |  | 37 | 212 |
| Kenya | N01 | FSW/MSW/MSM | 4,481 | 2 | 3,801 | 1.1 | 32 | 1,582 | 395 | 69.5 | 21 | 1,542 | 1,326 | 13 | 31 | 284 |
| Kenya | R01 | FSW/MSW/MSM | 4,408 | 3 | 1,087 | 29.6 | 30 | 2,430 | 464 | 123.7 | 23 | 2,768 | 125 | 121 | 237 | 55 |
| Kenya | R02 | FSW/MSW/MSM | 3,174 | 4 | 533 | 65.8 | 18 | 3,557 | 364 | 152.1 | 16 | 3,834 | 107 | 134 | 142 | 95 |
| Kenya | R03 | FSW/MSW/MSM | 1,318 | 8 | 522 | 65.6 | 25 | 2,420 | 466 | 75.4 | 20 | 2,694 | 132 | 89 | 119 | 95 |
| Kenya | R04 | FSW/MSW/MSM | 2,626 | 4 | 567 | 54.3 | 21 | 2,629 | 435 | 78.8 | 18 | 2,721 | 68 | 196 | 863 | 12 |
| Kenya | R05 | FSW/MSW/MSM | 436 | 28 | 830 | 37.8 | 30 | 2,033 | 450 | 102.3 | 22 | 2,126 | 83 | 174 | 276 | 42 |
| Kenya | C01 | MSM | 300 | 34 | 2,117 | 26.6 | 31 | 1,029 | 336 | 197.7 | 22 | 1,192 |  |  | 637 | 10 |
| Kenya | G | MSM | 2,030 | 6 | 46,513 | 0.3 | 25 | 1,422 | 382 | 78.7 | 23 | 1,597 |  |  | 328 | 17 |
| Kenya | B01 | MSW | 391 | 45 | 197 | 185.3 | 13 | 3,705 | 265 | 329.2 | 11 | 4,236 |  |  | 60 | 332 |
| Kenya | I01 | MSW | 3,256 | 2 | 602 | 44.5 | 64 | 1,839 | 375 | 157.8 | 21 | 2,191 |  |  | 187 | 43 |
| Kenya | Q01 | MSW |  |  | 994 | 15.5 | 42 | 914 | 1,119 | 12.9 | 40 | 794 |  |  | 592 | 28 |
| Malawi | B01 | FSW |  |  | 2,672 | 9.3 | 80 | 480 | 631 | 81.5 | 31 | 3,379 | 119 | 280 | 1,334 | 18 |
| Malawi | B02 | FSW |  |  | 3,463 | 5.6 | 72 | 437 | 583 | 80.0 | 25 | 4,071 | 88 | 343 | 1,380 | 16 |
| Malawi | B03 | FSW |  |  | 3,189 | 7.3 | 59 | 831 | 506 | 123.7 | 27 | 3,295 | 46 | 978 | 1,368 | 18 |
| Malawi | B04 | FSW |  |  |  |  | 60 | 1,045 | 613 | 172.2 | 38 | 2,339 | 185 | 161 | 699 | 37 |
| Malawi | B05 | FSW |  |  |  |  | 85 | 552 | 827 | 86.1 | 70 | 912 | 335 | 75 | 939 | 23 |
| Malawi | B06 | FSW |  |  |  |  | 122 | 378 | 713 | 96.5 | 43 | 2,438 | 162 | 183 | 983 | 24 |
| Malawi | B07 | FSW |  |  |  |  | 96 | 453 | 665 | 106.3 | 44 | 1,951 | 122 | 248 | 1,075 | 21 |
| Malawi | B08 | FSW |  |  |  |  | 87 | 492 | 843 | 81.3 | 61 | 1,298 | 453 | 54 | 1,107 | 20 |
| Malawi | B09 | FSW |  |  |  |  | 103 | 348 | 716 | 99.6 | 53 | 1,508 | 79 | 536 | 1,072 | 22 |
| Malawi | B10 | FSW |  |  |  |  | 86 | 521 | 1,056 | 42.7 | 57 | 1,681 | 1,790 | 12 | 967 | 22 |
| Malawi | B11 | FSW |  |  |  |  | 67 | 724 | 572 | 104.6 | 27 | 4,325 | 97 | 322 | 908 | 27 |
| Malawi | A01 | MSM/TGW |  |  |  |  | 64 | 793 | 4,383 | 5.8 | 40 | 3,054 |  |  | 18,029 | 1 |
| Malawi | A02 | MSM/TGW |  |  |  |  | 160 | 473 | 3,234 | 12.2 | 142 | 653 |  |  | 2,611 | 7 |
| Malawi | A03 | MSM/TGW |  |  |  |  | 133 | 328 | 3,472 | 8.0 | 78 | 1,471 |  |  | 4,217 | 4 |
| Malawi | A04 | MSM/TGW |  |  |  |  | 77 | 508 | 4,496 | 5.2 | 37 | 3,284 |  |  | 5,680 | 3 |

Notes: FY 2019 refers to U.S. Government fiscal year from October 1, 2018 to September 30, 2019, *FSW* female sex workers, *MSW* male sex workers, *MSM* men who have sex with men, *TGW* transgender women, *DIC* drop-in center, *PEP* post-exposure prophylaxis, *PrEP* pre-exposure prophylaxis, *HTS* HIV testing services, *ART* antiretroviral therapy, *STI* sexually transmitted infections, *SRH* sexual and reproductive health, *MSV* management of sexual violence, *UC* unit cost, *Q* number of services provided. The number of services provided during the year is displayed for PEP, HTS, STI services, SRH services, and MSV. For PrEP and ART, annual visits are shown.
